# Three-Dimensional Integrated Circuit (3D IC) Key Technology: Through-Silicon Via (TSV)

**DOI:** 10.1186/s11671-017-1831-4

**Published:** 2017-01-19

**Authors:** Wen-Wei Shen, Kuan-Neng Chen

**Affiliations:** Department of Electronics Engineering, National Chiao Tung University, Hsinchu, 300 Taiwan

**Keywords:** Through-silicon via (TSV), Three-dimensional integrated circuit (3D IC)

## Abstract

3D integration with through-silicon via (TSV) is a promising candidate to perform system-level integration with smaller package size, higher interconnection density, and better performance. TSV fabrication is the key technology to permit communications between various strata of the 3D integration system. TSV fabrication steps, such as etching, isolation, metallization processes, and related failure modes, as well as other characterizations are discussed in this invited review paper.

## Review

Three-dimensional integrated circuit (3D IC) and 2.5D IC with Si interposer are regarded as promising candidates to overcome the limitations of Moore’s law because of their advantages of lower power consumption, smaller form factor, higher performance, and higher function density [1–4]. To achieve 3D and 2.5D IC integrations, several key technologies are required, such as through-silicon via (TSV), wafer thinning, and handling, as well as wafer/chip bonding. Since TSV provides the advantages of shortening interconnection paths and thinner package size, it is considered as the heart of 3D integration. TSV formation is categorized into three types during 3D/2.5D IC process. When TSV is formed before CMOS processes, the process progression is defined as via first. In via middle flow, backend process only continues after the completion of TSV process. The final scheme is via last where TSV is fabricated from the front side or back side of wafer after completing the CMOS processes.

The choice of TSV schemes is based on the final application requirement in the semiconductor industry. TSV technology has been developed for many applications, such as MEMS, mobile phone, CMOS image sensor (CIS), bioapplication devices, and memory products. Thus, a number of studies have been conducted on the manufacturing of TSV. In current status, with the relatively high fabrication cost, TSV implementation in 3D IC and advanced packaging applications is not generally implemented yet [5, 6]. In this paper, we review the important manufacturing processes of TSV and related failure modes when TSV has a smaller diameter and higher aspect ratio. Furthermore, TSV fabrication has various important processes, including via formation by deep reactive ion etching (DRIE), lining with dielectric layer, barrier and seed layers, via filling, chemical mechanical polishing (CMP), and Cu revealing process. Each key technique will be introduced in detail in the following sections.

## TSV Etching

**Fig. 1:**
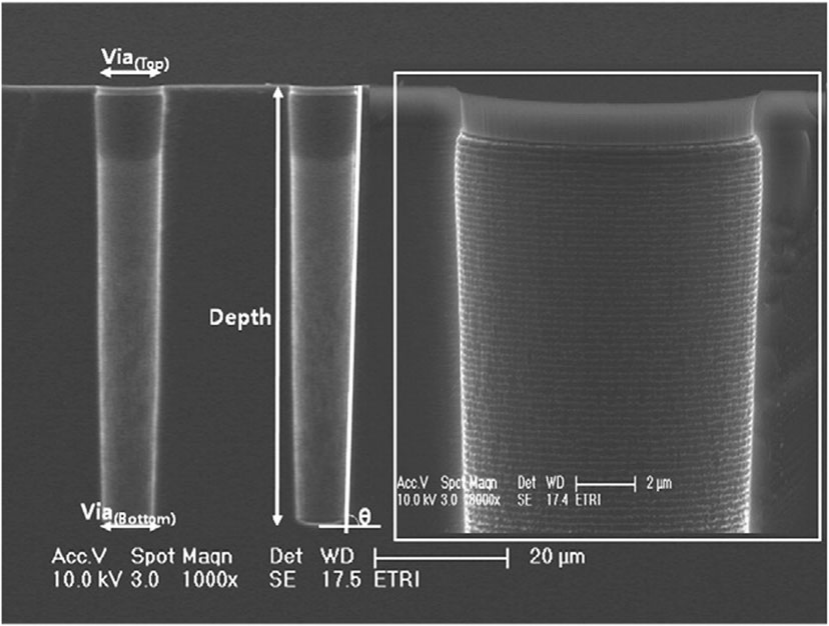
Si etching result of 10-μm via hole using Bosch process and structure definition of silicon via [8]

TSV etching is employed as a key fabrication module in 3D integration technologies while the widely used Bosch process is preferred for deep Si etching. Bosch etching process has a high etching rate of 5~10 μm/min, selectivity to photoresist of 50–100, and up to 200 of an oxide mask. The process is executed by the following steps: (1) Si etching with the utilization of SF_6_ as an etchant; (2) in combination with C_4_F_8_ gas, generates good passivation films for preventing lateral Si during next Si etching step; and (3) further etching of passivation and Si layer in SF_6_ plasma by using directional ion bombardment to form a deep etching depth. Then, the passivation layer is cleaned through O_2_ and Ar plasma. The schematic of Bosch of Fig. 1 shows the structural definition of 10 μm TSV [7, 8]. However, it inevitably causes sidewall scalloping roughness which may induce poor step coverage of following processes, resulting in electrical leakage and reliability issues. Developing the right amount of sidewall roughness in TSV etching is a matter of balancing the etching and passivation process during the time-multiplexed deep silicon etching [9]. The sidewall scalloping impacts dielectric, barrier, and Cu seed layer coverage by enhancing the voids in the TSV; thus, the sidewall scalloping needs to be minimized as the size of TSV reduces.

## TSV Dielectric Layer

**Fig. 2:**
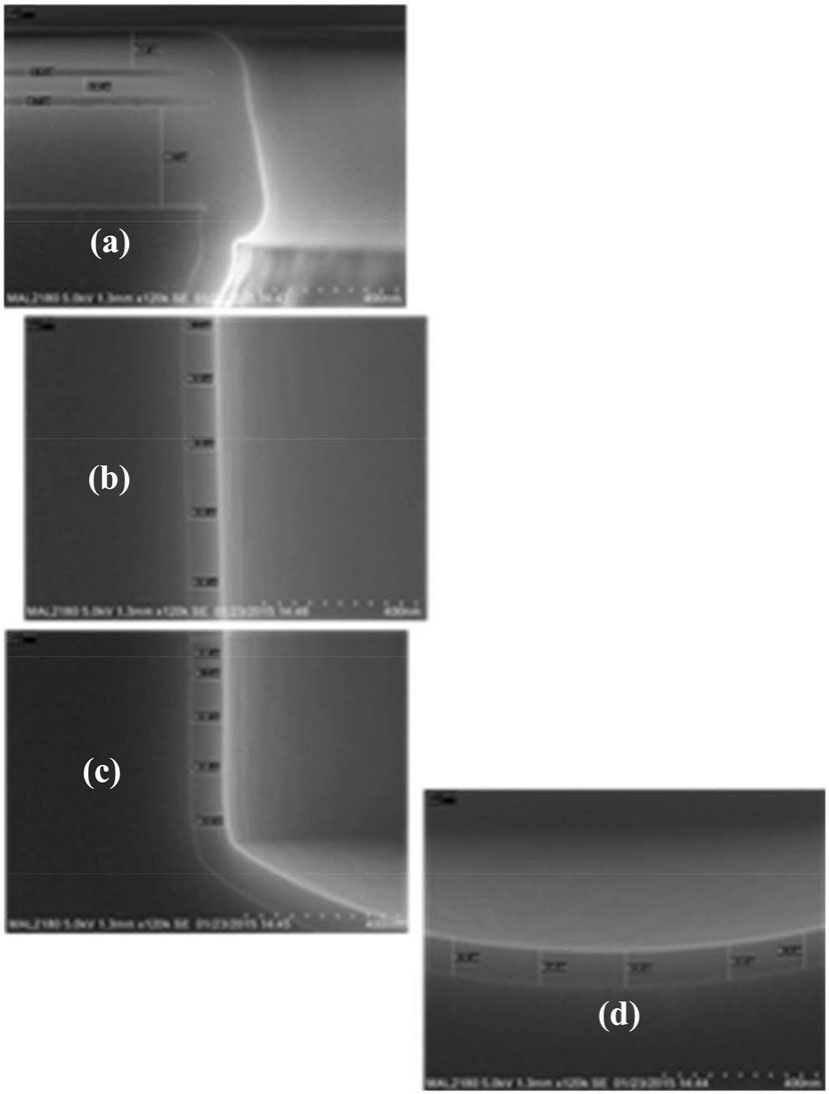
Cross-sectional SEM images for a 3 × 50 μm TSV after ALD dielectric oxide layer deposition. **a**–**d** 91∼95-nm-thick film around TSV [11]

Metal-filled TSV needs a dielectric layer for sufficient electrical isolation to the surrounding Si substrate. Process requirements for a dielectric layer include good step coverage and uniformity, no leakage current, low stress, higher breakdown voltage, and processing temperature limitations due to different TSV integration [10]. SiO_2_ or Si_3_N_4_ is usually used as the dielectric layer in plasma-enhanced chemical vapor deposition (PECVD) or sub-atmospheric chemical vapor deposition (SACVD) for TSV. However, when diameter of TSV is smaller than 3 μm, the dielectric layer is suitable to be deposited by atomic layer deposition (ALD). ALD has several advantages such as lower thermal budget, better step coverage than existing processes, scalability without requiring surface treatment prior to dielectric deposition, and reduced CMP processing time of TSV due to the thinner dielectric layer. The conformal coverage of 100-nm ALD dielectric oxide layer is deposited around TSV with the dimensions of 3 × 50 μm, and the thickness of the oxide layer on sidewall and bottom are approximately 95 nm, as shown in Fig. 2 [11]. The aspect ratio (AR) of TSV is 17, and the result demonstrates an excellent profile as a dielectric layer for miniature TSV applications.

## TSV Barrier and Seed Layers

The immediate following process is the deposition of barrier layer to prevent the diffusion of Cu atoms from Cu TSV during annealing processes that require temperature at 400 °C. Besides, the barrier acts as an adhesion layer between the dielectric layer and the Cu layer. The common materials that are used as barrier layers are Ti, Ta, TiN, and TaN; physical vapor deposition (PVD), CVD, and ALD are the methods implemented in general depending on the dimensions of the TSV used. The metal barrier layers are deposited through PVD, such as Ta and Ti. This approach has the benefits of low temperature during process but suffers poor step coverage easily for high aspect ratio TSV (>10:1) [12]. Thus, a thicker metal barrier is deposited to overcome poor step coverage but increases the production cost. TiN or TaN barrier layers can be deposited using CVD method, which has the advantage of good uniformity but requires high processing temperature.

**Fig. 3:**
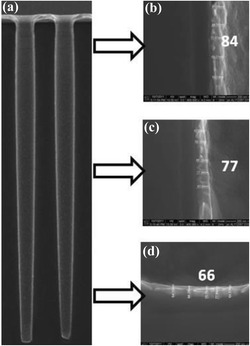
Cross-sectional SEM micrograph images of 2 × 30 μm TSV after alkaline Cu seed deposition prior to ECD fill. **a** Overview, **b** top, **c** middle, and **d** bottom of the TSV. An excellent conformality, in the range of 80%, is obtained for the alkaline Cu seed in the TSV region of 4-μm pitch [13]

In the following process, Cu seed is usually deposited in the TSV by adopting PVD method. In IMEC study [13], by using ALD TiN as a barrier, an approximate uniformity of 80% has been achieved for metalizing the 2 × 30 μm TSV (aspect ratio = 15). Then, a continuous and highly conformal alkaline seed layer is successfully deposited along the TSV sidewalls and bottom. In this demonstration, the alkaline Cu seed layer has been efficiently replaced by the PVD Cu prior bottom-up filling, as shown in Fig. 3. Results of subsequent void-free filling of TSV have been obtained on the wafer. Cost and thermal budget reduction of the barrier and seed layer processes are the key challenges for the application of Cu-TSVs.

**Fig. 4:**
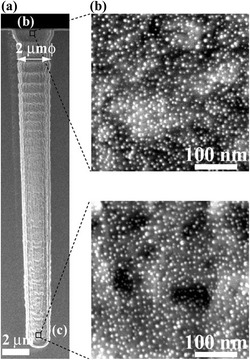
Cross-sectional SEM images after adsorption of Pd-NPs on the TSV sidewall (2 × 24 μm). **a** Overview, **b** top, and **c** bottom of the TSV [15]

**Fig. 5:**
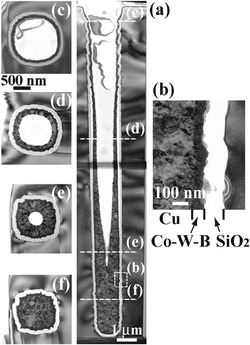
Cross-sectional TEM images of an electroless Cu/Co–W–B bilayer in a TSV. **a** Overview, **b** vertical cross-sectional image of sidewall, and **c**–**f** horizontal cross-sectional images at various depths [15]

TSV process conducted after the back-end-of-line interconnect is a concern of the process temperature for the device reliability test. Thus, an all-wet process at low temperature is performed to form electroless deposition of barrier and Cu seed layers for high aspect ratio TSV. Electroless depositions of Co–W–B and Cu as barrier/seed layers are achieved by using Au nanoparticles (Au-NPs) or Pd nanoparticles (Pd-NPs) as a catalyst [14–16]. Morphologies from different positions of one TSV after the adsorption of Pd-NPs at room temperature for 3 h are shown in Fig. 4. Pd-NPs are deposited uniformly throughout the 2 × 24 μm TSV, and no Pd-NP agglomeration is observed. An electroless Cu/Co–W–B as the following process can be seen in the cross-sectional TEM images of Fig. 5. It was achieved by using Pd-NPs as a catalyst throughout the TSV. Even though there is a periodic scalloping of the TSV sidewall, a continuous and uniform Co–W–B film with a thickness of 60 nm at 60 °C is deposited into the TSV successfully. By displacement plating at 70 °C, an electroless Cu layer is deposited directly on the Co–W–B layer. The diffusion flux of inhibitors at the bottom of the TSV is lower than that at the top; hence, the Cu seed layer from electroless deposition is thicker at the bottom of the TSV than at the top. Although all-wet barrier and seed layers require low processing temperature with great step coverage, more experiments are needed to prove its reliability.

## TSV Filling

TSV filling has three plating methods: conformal plating [17, 18], sealing bump with bottom-up plating [19, 20], and super-conformal plating [21–25]. The plating methods are based on various 3D integration applications. In general, TSV structures have a typical cylinder with depth between 10 and 200 μm. The TSV depth is defined by the required thickness during chip or wafer staking, and the aspect ratio is established through fabrication of the dielectric layer/barrier/seed/filling process. Although there are many different TSV geometries for 2.5D and 3D integration application, they can be summarized into three general types as stated in Table 1 [26].

**Table 1:** General types of TSV [26]

Application	Plating	Depth	Diameter	Aspect ratio
Image sensor	Conformal	50 to 100	30 to 50	1 to 3
Interposer	Full-fill	50 to 150	20 to 30	4 to 8
Device	Full-fill	20 to 60	2 to 10	5 to 15

### Conformal Plating

**Fig. 6:**
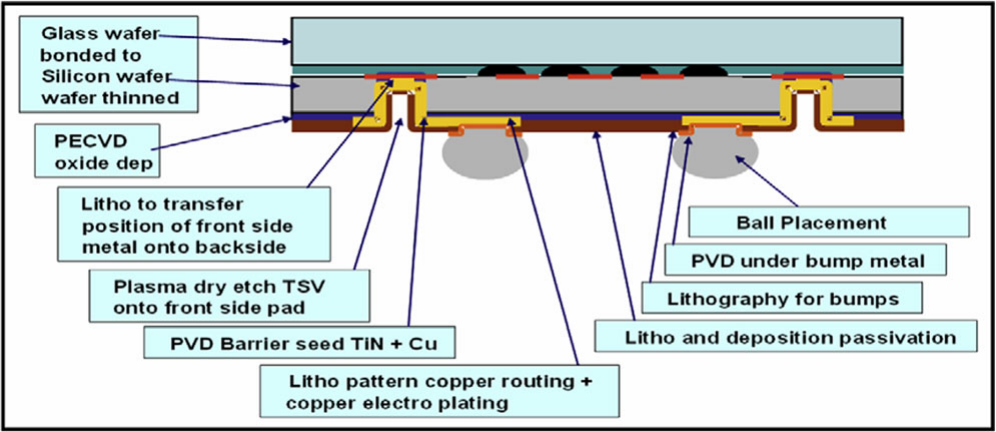
Cross section of the CIS developed with TSV [27]

**Fig. 7:**
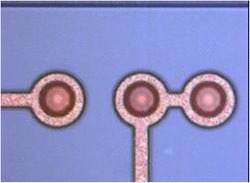
Optical image of TSV after Cu ECD and seed layer etching [17]

**Fig. 8:**
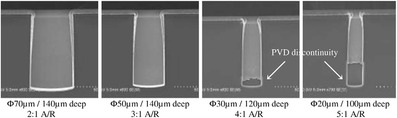
TSV cross section of different aspect ratios via after barrier/seed deposition and Cu conformal plating [18]

Conformal Cu plating is similar to Cu pattern plating for redistribution layers (RDL) or wafer-level chip scale package (WLCSP) wiring in resist masks, and the application is suitable for low aspect ratio partially TSVs. As an example of CIS application, its main processes can be seen in Fig. 6, including the deep RIE of silicon to reach the CMOS metal layer, oxide isolation of the via, the barrier and seed PVD deposition, and finally the Cu conformal plating for RDL [27]. Cu thickness ranging from 5 to 10 μm is grown in a resist mask structure that deposits the topography of TSVs and wiring patterns on top of the silicon, as shown in Fig. 7 [17]. Figure 8 shows the cross-sectional scanning electron microscope (SEM) images of different aspect ratios via which increases from AR of 1 to AR of 5 after Cu conformal plating. However, the applications are limited to AR of 3 due to the discontinuity of the Cu seed layer [18].

### Sealing Bump with Bottom-up Plating

**Fig. 9:**
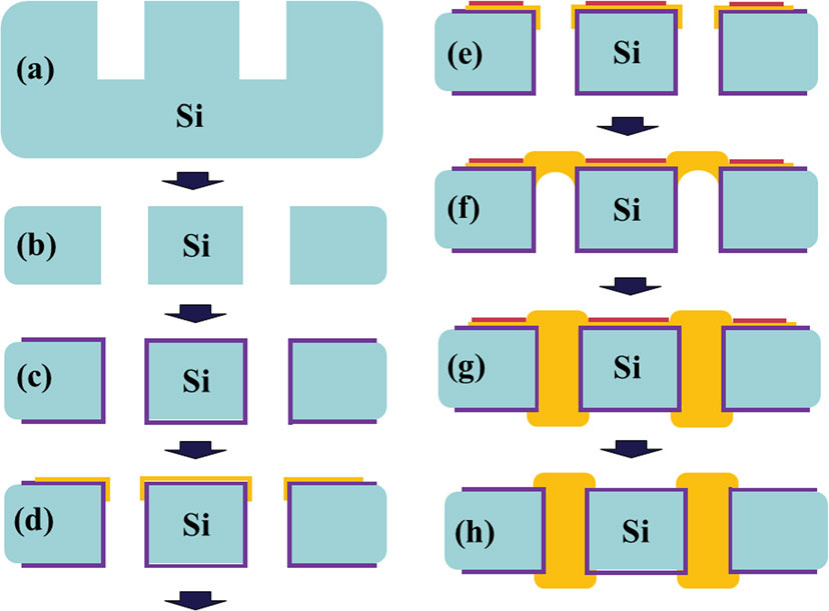
Process flow of proposed sealing bump bottom-up plating approach. **a** TSV etching. **b** Thinning. **c** Oxide insulation. **d** Seed layer deposition. **e** Photoresist patterning. **f** Bump sealing formation. **g** TSV and bump plating. **h** Final etching [20]

**Fig. 10:**
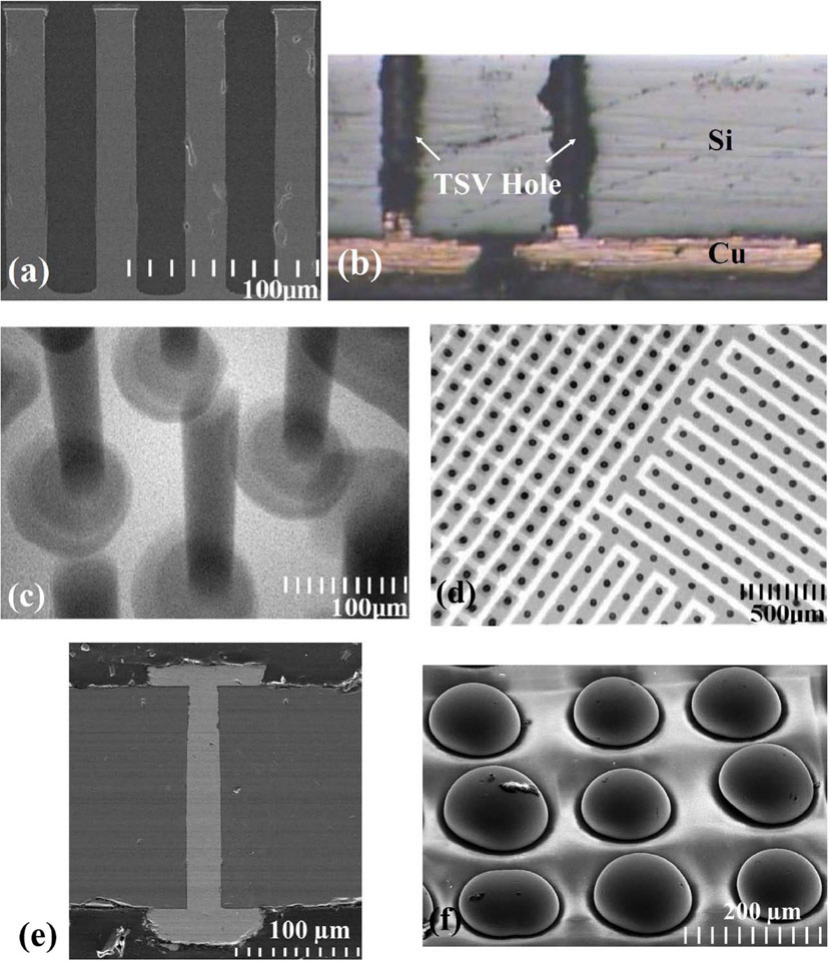
**a** Dry etching profile of 25 μm via. **b** Sealing bumps fabricate before TSV filling. **c** Void-free filled TSVs by X-ray inspection. **d** The *black dots* are Cu TSVs; the *white area* is the SiO2 region; the *gray-colored area* is the metal lines. **e** TSV cross section with Cu bumps on both sides. **f** Final structures of Cu bumps with TSVs [20]

One of the advantages of the bottom-up TSV approach is the ability to avoid seams or voids during via filling [28, 29]. Furthermore, the bottom-up process is suitable for via last scheme. It usually requires temporary bonding or attaching technology with Cu seed layer at the bottom to complete the via filling process. The removal of handling carrier or attached metal may lead to extra cost and reliability issue; thus, a novel approach of sealing bump before Cu TSV filling based on bottom-up plating process is proposed as shown in Fig. 9 [20]. SEM, optical microscope, and X-ray analysis are observed to guarantee no defects after bottom-up plating by the approach proposed in Fig. 10. The TSV and bump structure are fabricated in a one-step plating process to simplify the fabrication flow allowing it to be applicable by via last approach in the 3D integration scheme.

### Super-conformal Plating

**Fig. 11:**
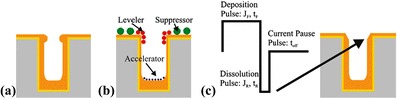
Principle TSV filling by super-conformal plating. **a** Non-optimized DC plating. **b** Additive approach. **c** PPR current waveform [30]

Super-conformal Cu filling is adapted over a wide range of dimensions, from near damascene scale features to large features used for interposer and device applications. The general requirement shows no seams or voids within the TSV through X-ray observation while the Cu overburden and barrier layer are removed by CMP. Figure 11 shows the principle of TSV filling, including plating recipe characteristic and organic additives properties [30]. The plating recipe establishment is a critical factor for TSV filling due to pinch-off issue in standard DC plating, as shown in Fig. 11a.

**Fig. 12:**
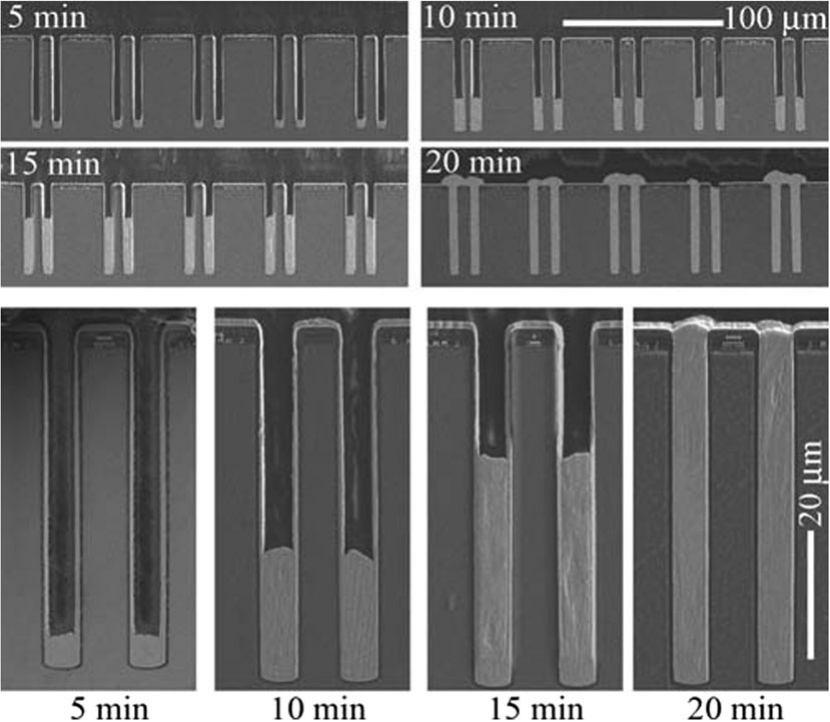
TSV cross-sectional images showing the progression of bottom-up Cu filling of ring-shaped vias while almost negligible deposition has occurred on the neighboring free surface. The top four images demonstrate the uniformity of filling within the via arrays [39]

TSV filling chemical bath typically uses three organic additives, including suppressor, accelerator, and leveler [31–35]. A slow diffusing and rapidly adsorbing suppressor, such as polyethylene glycol (PEG), adsorbs primarily at the flat surface. A fast diffusing accelerator, such as bis-(3-sulfopropyl)-disulfide (SPS), penetrates the via and enhances the deposition rate. A slow diffusing leveler, such as Janus Green B (JGB), can de-activate the accelerator and distribute along the rim. Adsorption results of variable kinetics and additives deposition are shown in Fig. 11b. A periodic pulse reverse (PPR) current waveform is applied to prevent TSV premature closure for the Cu filling. Four parameters are adopted to establish plating recipe, including reverse pulse time (tR), current pause time (toff), forward pulse time (tF), and corresponding current densities’ (jF, jR) constant, as shown in Fig. 11c [36, 37]. Furthermore, the three-step PPR current waveform is suggested to reduce the Cu-filling time and to reduce the amount of defects in the TSV filling [38]. The progression of bottom-up Cu filling is shown in Fig. 12, which indicates the 8 × 56 μm TSV arrays after 5, 10, 15, and 20 min of Cu filling in the CuSO_4_ + H_2_SO_4_ + Cl^−^ polyether suppressor system. The void-free feature filling is observed after 20 min [39].

However, filling of high aspect ratio of TSVs takes a long time due to the usage of pulse reverse current that is depleted to Cu ions on the via sidewall. Thus, shortening the TSV filling time is necessary for 3D integration. There are four types of optimization approaches to enhance the filling efficiency, including anode position optimization, a multi-step TSV filling process, additive concentration, and plating current density optimization [40]. Finally, CMP is used to remove the Cu overburden as well as barrier layer from the wafer surface. In general, this technology requires two steps. The first step is to remove the thick blanket Cu with dimples or recesses after TSV filling, and it stops at the barrier layer. The second step removes the barrier layer, stopping at the dielectric layer. Different slurries with selectivity are used to realize insulation well, minimize topography, and avoid defects like dishing and erosion [41].

## TSV Cu Revealing

**Fig. 13:**
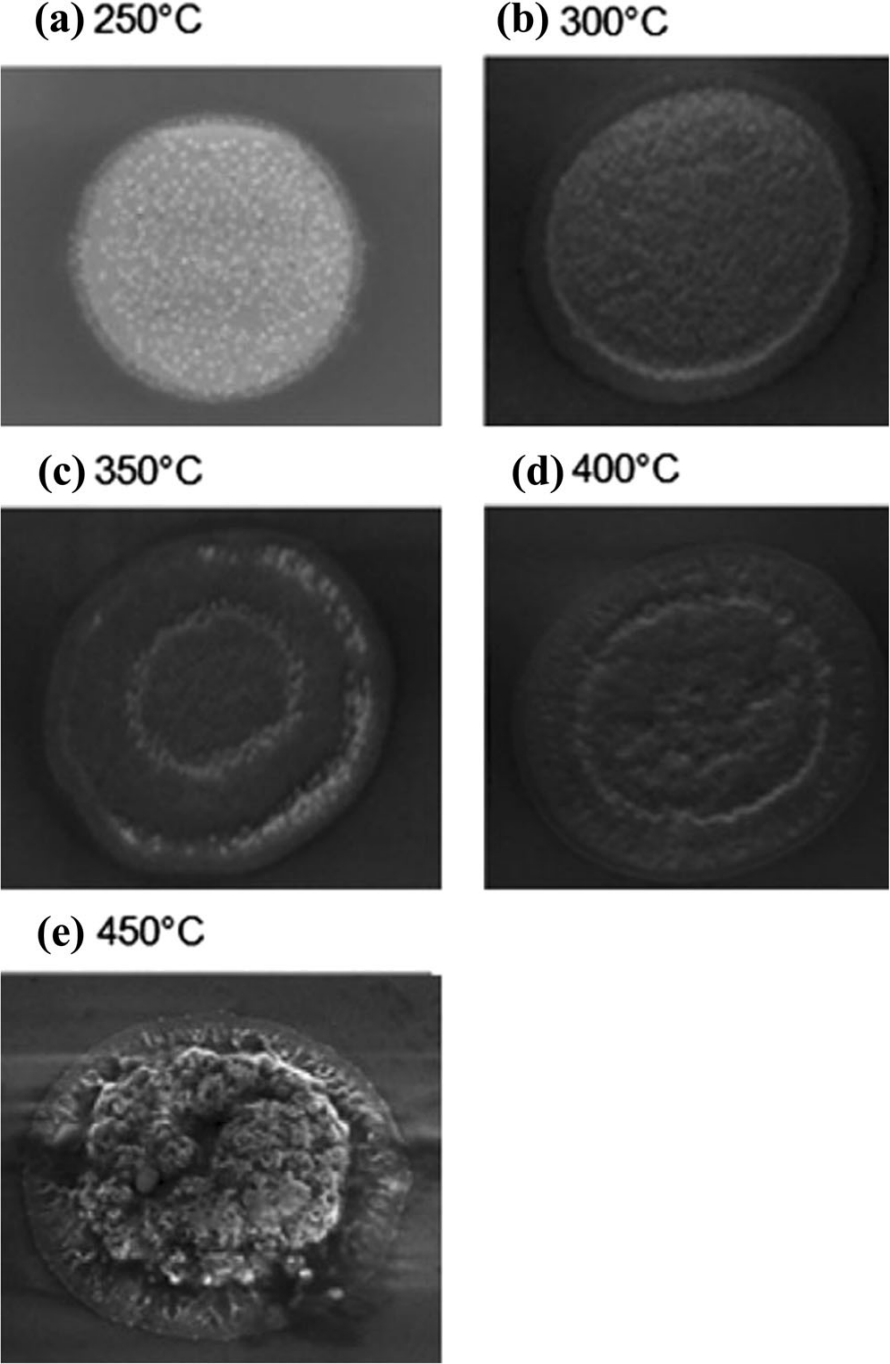
Top view SEM micrographs of TSVs, showing the extent of protrusion at various annealing conditions ranging from annealing temperature *T* = 250 to 450 °C [44]

Another key process is the TSV extrusion or TSV pumping issue due to the mismatch in coefficient of thermal expansion (CTE) between the Cu material and Si substrate [42, 43]. The thermal expansion of copper is 17.6 ppm/°C, which is higher than silicon of 2.6 ppm/°C, inducing several reliability issues such as cracking and delamination of the dielectric layer. The influence of annealing process was experimented on with samples prepared to a range of annealing processes with several conditions. Figure 13 indicates SEM micrographs of the protruding 5 × 50 μm TSVs in range of 250 to 450 °C for 30 min, respectively, demonstrating the shape of the protrusion due to the annealing temperature. The Cu protrusion starts from annealing temperature at 350 °C, and it bulges upward at 450 °C as shown in Fig. 13e. The Cu protrusion phenomenon has two possible mechanisms. The first mechanism is the plastic deformation of the Cu material that expands vertically during annealing. The second mechanism is due to diffusive creep when the stress distribution is not uniform within the TSV [44]. It is necessary to reduce the silicon stress through suitable pre-annealing after the TSV electroplating process, and then, CMP is used to remove Cu overburden and linearize the TSV.

## TSV Failure Modes

**Fig. 14:**
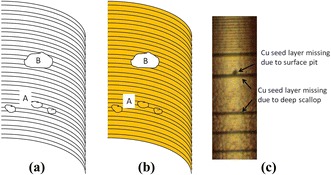
Mechanism causing the Cu seed layer missing due to sponge-like defects and deep scallops. **a** After Si etch. **b** After Cu seed layer deposition. **c** Microscopic image of the Cu seed layer deposited at 60 μm × 250 μm TSV [45]

**Fig. 15:**
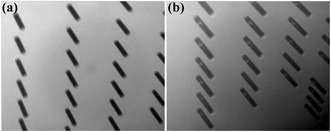
X-ray images of TSVs after Cu electroplating. **a** TSVs without Cu filling failure. **b** TSVs with Cu filling failure caused by the Cu seed layer loss due to the sponge-like defects at 30 μm × 150 μm TSV [45]

TSV-related failure modes are categorized into three major regions: Si etch related, Cu seed layer related, and Cu electroplating related [45]. If there are some issues in the TSV process integration, several failure modes can be observed as voids after Cu electroplating. Since TSVs are dry etched with Bosch process as previously mentioned, there are several related Si etch defects resulting in Cu seed layer loss, including bottom corner notch, Si grass at the bottom of the TSV, surface roughness, and sponge-like defects, as shown in Fig. 14. TSVs with Cu filling failure caused by the sponge-like defects at 30 μm × 150 μm TSV, as shown in Fig. 15, may cause electrical disconnection as well. The second failure mode can be from the oxidation of Cu seed layer and poor Cu seed layer step coverage.

**Fig. 16:**
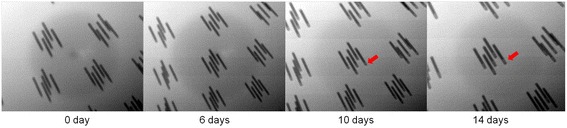
X-ray images showing queue time after Cu seed layer deposition at 10 μm × 100 μm TSVs [45]

With the impact of the Cu seed layer oxidation on TSV Cu filling, the voids begin to form at the top area of TSV after 10 days from the PVD-Cu seed deposition [45]. It demonstrates that Cu oxide enhances the terminal effect by reducing the Cu seed layer step coverage, as shown in Fig. 16. Lastly, it is important to optimize the chemical concentration of the three additives and current density to avoid filling failure for the mentioned Cu electroplating-related region. Therefore, TSV formation without the voids can be achieved by improving related failure modes.

## Conclusions

This review paper summarizes various TSV fabricated technologies for 3D integration, including the processes development, Cu filling methods of various applications, and filling failure modes. The dielectric, barrier, and seed layers are developed to overcome Si sidewall scalloping roughness and solve discontinuity of Cu seed through wet process with high aspect ratio TSV. Cu TSV filling has three plating methods: conformal plating, sealing bump with bottom-up plating for void-free filling and simplicity of fabrication flow, and super-conformal plating that is used for interposer and device applications. Furthermore, TSVs with voids may also lead to electrical failure and reliability issues, and the root causes are also reported.

## Abbreviations

3D IC: Three-dimensional integrated circuit
ALD: Atomic layer deposition
AR: Aspect ratio
Au-NPs: Au nanoparticles
CIS: CMOS image sensor
CMP: Chemical mechanical polishing
CTE: Coefficient of thermal expansion
DRIE: Deep reactive ion etching
JGB: Janus Green B
Pd-NPs: Pd nanoparticles
PECVD: Plasma-enhanced chemical vapor deposition
PEG: Polyethylene glycol
PPR: Periodic pulse reverse
PVD: Physical vapor deposition
RDL: Redistribution layers
SACVD: Sub-atmospheric chemical vapor deposition
SEM: Scanning electron microscope
SPS: Bis-(3-sulfopropyl)-disulfide
TSV: Through-silicon via
WLCSP: Wafer-level chip scale package
